# Treatment of malaria from monotherapy to artemisinin-based combination therapy by health professionals in urban health facilities in Yaoundé, central province, Cameroon

**DOI:** 10.1186/1475-2875-8-176

**Published:** 2009-07-29

**Authors:** Collins Sayang, Mathieu Gausseres, Nicole Vernazza-Licht, Denis Malvy, Daniel Bley, Pascal Millet

**Affiliations:** 1 Department of Tropical Medicine, Centre René Labusquière, University of Bordeaux 2, 146 rue Léo Saignat 33076 Bordeaux Cedex France; 2 Department of Anthropology, DESMID – UMR 6012 Espace, 1, rue Parmentier – 13200 Arles, France

## Abstract

**Background:**

After adoption of artesunate-amodiaquine (AS/AQ) as first-line therapy for the treatment of uncomplicated malaria by the malaria control programme, this study was designed to assess the availability of anti-malarial drugs, treatment practices and acceptability of the new protocol by health professionals, in the urban health facilities and drugstores of Yaoundé city, Cameroon.

**Methods:**

Between April and August 2005, retrospective and current information was collected by consulting registers and interviewing health practitioners in urban health facilities using a structured questionnaire.

**Results:**

In 2005, twenty-seven trade-named drugs have been identified in drugstores; quinine tablets (300 mg) were the most affordable anti-malarial drugs. Chloroquine was restricted to food market places and no generic artemisinin derivative was available in public health centres. In public health facilities, 13.6% of health professionals were informed about the new guidelines; 73.5% supported the use of AS-AQ as first-line therapy. However, 38.6% apprehended its use due to adverse events attributed to amodiaquine. Malaria treatment was mainly based on the diagnosis of fever. Quinine (300 mg tablets) was the most commonly prescribed first-line anti-malarial drug in adults (44.5%) and pregnant women (52.5%). Artequin^® ^was the most cited artemsinin-based combination therapy (ACT) (9.9%). Medical sales representatives were the main sources of information on anti-malarials.

**Conclusion:**

The use of AS/AQ was not implemented in 2005 in Yaoundé, despite the wide range of anti-malarials and trade-named artemisinin derivatives available. Nevertheless, medical practitioners will support the use of this combination, when it is available in a paediatric formulation, at an affordable price. Training, information and participation of health professionals in decision-making is one of the key elements to improve adherence to new protocol guidelines. This baseline information will be useful to monitor progress in ACT implementation in Cameroon.

## Background

The greatest number of people exposed to stable transmission of *Plasmodium falciparum *lives in sub-Saharan Africa. In most of the endemic countries, access to appropriate health care is limited and the problems related to drug availability and resistance are associated with an increasing mortality rate [1]. In sub-Saharan Africa, chloroquine has been the base-line drug for the treatment of mild malaria cases, followed by sulphadoxine-pyrimethamine as the cheapest second-line drug. However, recent reports showed that the clinical efficacy of both drugs has significatively declined [2].

Between 1997 and 2004, twenty-five surveys were conducted in Cameroon in order to evaluate the therapeutic efficacy of first-line and second-line anti-malarial treatments, using the standardized protocol of the World Health Organization (WHO). Results indicated that chloroquine was no longer effective in southern and central provinces and presented a therapeutic failure rate greater than 25% [1]. Sulphadoxine-pyrimethamine (SP) was associated with failure rates ranging from 8.6% to 14.1%. Amodiaquine remained effective in the entire country with a failure rate estimate of approximately 4%, although the drug was used as first-line anti-malarial therapy from 2002 to 2004.

Recently, WHO proposed modifications of endemic countries guidelines, changing from monotherapy to artemisinin-based combination therapy (ACT). In view of this, and after a scientific consensus meeting held in January 2004, the National Malaria Control Programme of Cameroon announced that amodiaquine will be replaced by the combination artesunate-amodiaquine (AS/AQ) (artesunate 4 mg/kg/day, amodiaquine 10 mg/kg/day). This combination will be used as first-line therapy for three days for the treatment of uncomplicated malaria [3,4]. These guidelines clearly stated that (i) injectable quinine or injectable artemether would be administered only in case of drug failure or severe malaria, and (ii) artemisinin derivatives should not be given to pregnant women during the first trimester of gestation and quinine remained the recommended treatment for any malaria cases during pregnancy. The new treatment guidelines are based on a clinical (fever) and laboratory (thick blood smear) diagnosis procedure and recommend an evaluation of treatment efficacy by health professionals, four days post-treatment [4].

Recommended drugs are provided through the National Centre for the Provision of Essential Drugs (CENAME), who signed an agreement with religious health institutions, non-lucrative health units, trade-union of pharmacists, and wholesalers of drugs.

Implementation of new treatment guidelines based on the use of ACT requires the full participation of practitioners from all health institutions. At the time of the study, generic artemisinin derivatives were not available at CENAME. Nevertheless various trade-named anti-malarial drugs were found in private drugstores, especially in urban areas. The present study reports anti-malarial drug prescribing practices of medical doctors and nurses, in urban health facilities in Yaoundé city, central province of Cameroon, and analyses attitudes and practices one year after new treatment guidelines based on AS/AQ were approved by Cameroonian authorities.

## Methods

### Sites of investigations

This study was conducted in the urban setting of Yaoundé, in the central province of Cameroon. A total of 23 health facilities were investigated including two general hospitals, three district hospitals, eight public health centres, 10 private health services and six private drug stores.

### Population

A total of 132 health professionals were interviewed including 31.8% medical doctors, 60.6% nurses and 7.6% health assistants. 52.3% worked in hospitals and 68.9% of them belonged to public institutions.

### Data collection

All information on anti-malarial drugs available in public and private health facilities was pooled in order to investigate drug availability. Information about practices and behaviours was collected from health professionals using a structured questionnaire, previously tested and implemented in two health centres. The questionnaire was divided into four sections including: 1) evaluation of knowledge and attitudes of medical practitioners according to treatment guidelines; 2) current use and regimen of anti-malarial drugs as first-line and second-line therapy; 3) knowledge and use of artemisinin derivatives; and 4) sources of information on malaria and anti-malarial drugs. Additional information was collected from the health facility's documents and registers.

### Data analysis

Responses from interviews were numerically coded and analysed using Epi-Info version 6.04. Treatment cost defined in US$ are expressed as a mean. Differences in proportion were analysed using chi-square test, when appropriate and significance was set at p < 0.05. This protocol was approved by the operational research board of the Ministry of Public Health, in Cameroon.

## Results

### Availability of anti-malarial drugs

In Yaoundé, up to August 2005, private drugstores could acquire thirty-two generic-named drugs from CENAME. The only anti-malarial drug accessible was quinine (tablets 300 mg), representing the first-line drug sold in private and public sectors (representing 7% of the total drug consumption). In addition, three artemisinin-based combinations (Arsucam^®^, Coartem^® ^and Artequin^®^) and twenty-seven trade-named artemisinin derivatives were available in private drugstores. Chloroquine was mainly available on food market places.

### Knowledge on new treatment guidelines

13.6% of the 132 health professionals were informed about treatment guidelines and knew that AS/AQ was the recommended drug for the treatment of uncomplicated malaria, in Cameroon. Four of them (3.0%) reported having the document in their office, at the time of investigation.

### Attitudes related to AS/AQ and treatment guidelines

About three quarters of health professionals supported the use of AS/AQ as first-line therapy in the new treatment protocol, while others were in favour of quinine administration. However, health professionals mentioned that restrictions in the use of AS/AQ were based on the following parameters: mild to severe adverse events attributed to amodiaquine (38.6%); risk of non-observance of the treatment related to the high number of tablets intake (24 pills total for the adult regimen)(28.8%); absence of paediatric formulation (suppositories and oral solutions) (21.9%); drug cost (6.1%); clinical failures associated with artesunate used in monotherapy for three days (4.6%).

### Practices regarding malaria diagnosis

The first-line treatment of malaria was administered following a clinical diagnosis based upon the presence of fever by three quarters of the practitioners. In addition, a thin or thick blood smear examination could be performed before treatment; however laboratory results were obtained after drug prescription and administration.

### Anti-malarial drugs used as first-line treatment

Quinine (300 mg tablets) was the most commonly prescribed anti-malarial drug in adults (44.5%) and pregnant women (52.5%) (Table 1). For children, 62.8% of the prescribers were in favour of oral suspensions of amodiaquine alone while 4.6% of the physicians administered the recommended AS/AQ (Arsucam^®^) combination therapy to adults and 1.5% to children. Artemether-lumefantrine (Coartem^®^) was the most used form of ACT (8.3% in adults). Health workers prescribed suppositories of artesunate (Plasmotrim^®^) (3.8%) and tablets of dihydroartemisinin (Cotecxin^®^) (2.3%) to pregnant women, but the prescription of AS/AQ (Arsucam^®^) was not reported in this group. There were no significant differences between prescription of proper dosage and drug regimens.

**Table 1:** Anti-malarial drugs used as first-line treatment by medical practitioners

***Drugs prescribed by 132 clinicians***	***Adults*** ***N *= *128(%)***	***Pregnant women n = 124 (%)***	***Under five children*** ***n *= *126 (%)***
AS/AQ	4.6	0.0	1.5
Quinine tablets	44.5	52.3	15.9
Amodiaquine	20.4	28.0	62.8
Artemether-lumefantrine	8.3	0.0	2.4
Artesunate-mefloquine	1.5	0.0	0.8
Artesunate suppositories	7.6	3.8	5.3
Dihydroartemisinin	5.3	2.3	4.6
Halofantrine	0.0	0.0	1.5
Sulphadoxine/Pyrimethamine	5.8	3.8	0.0
Pyrimethamine alone	0.0	0.8	0.0
Injectable quinine	3.0	9.0	4.5
Injectable artemether	0.8	0.0	2.0

### Anti-malarial drugs used as second-line treatment

In case of clinical failure, about 90% of the practitioners intended to prescribe a laboratory diagnosis before administration of a second-line treatment. The proportion of second-line therapy showed that injectable quinine (intramuscular) was the first-line anti-malarial drug prescribed in adults (43.2%), pregnant women (53.7%) and children (36.4%) (Table 2). Intra-muscular injectable formulations were prescribed, with a mean treatment schedule of two injections per day for two days. The second drug of choice was quinine in the form of tablets of 300 mg, in adults (20.4%) and pregnant women (19.7%), and as an oral solution in children (13.6%). According to guideline recommendations, injectable artemether was prescribed in adults (3.0%), pregnant women (1.5%) and under five children (3.8%). Other artemisinin derivatives were prescribed as second-line drugs, such as artesunate suppositories (18.1%), Cotecxin^® ^(10.6%), Artequin^® ^(9.8%), Coartem^® ^(3%), Artesiane^® ^(2.3%). Only one health professional prescribed antibiotics associated with anti-malarial drugs, to cover for other infections.

**Table 2:** Anti-malarial drugs used as second-line treatment by medical practitioners

***Drugs prescribed by 132 clinicians***	***Adults*** ***N *= *124 (%)***	***Pregnant women n = 113 (%)***	***Under five children*** ***N *= *122 (%)***
Injectable quinine	43.2	53.7	36.4
Injectable Artemether	3.0	1.5	3.8
AS/AQ	2.3	3.0	2.3
Artesunate-mefloquine	9.8	0.8	3.8
Artemether-lumefantrine	3.0	0.0	0.8
Quinine (oral)	20.4	19.7	13.6
Amodiaquine	0.8	1.5	6.0
Artesunate suppositories	10.6	5.3	18.1
Dihydroartemisinin	3.0	7.6	10.6
Artemether, oral	0.0	1.5	2.3
Halofantrine	0.8	0.0	0.0
Injectable Sulphadoxine/Pyrimethamine	2.3	3.8	1.5
Sulphadoxine/Pyrimethamine	0.0	0.8	0.0
Quinine + antibiotics	0.0	0.8	0.8

### Attitudes and practices related to artemisinin derivatives

The evaluation of knowledge about artemisinin derivatives indicated that 5.3% of the nurses (7 nurses) reported receiving no information related to these drugs (Table 3). On the other hand, trade-named anti-malarial drugs containing artemether (42.4%) or dihydroartemisinin (19.7%) were the well known and the most frequently cited drugs. The first combination therapy being Artequin^® ^(9.9%) and Coartem^® ^(7.6%) followed by the less cited Arsucam^® ^(7%), a co-formulation of artesunate-amodiaquine. 84.1% of the health professionals knew that these anti-malarial drugs should not be administered in pregnant women during the first trimester of gestation.

**Table 3:** Artemisinin derivatives known by medical practitioners in urban zones

***Artemisinin derivatives***	***Medical practitioners*** ***(n = 132), Percent***
	**Informed (n = 125)**
***Monotherapies***	
Artesunate suppositories	(56) 42.4
Dihydroartemisinin	(26) 19.7
Artemether	(11) 8.3
***Bitherapies (ACT)***	
Artesunate-mefloquine	(13) 9.9
Artemether-lumefantrine	(10) 7.6
Artesunate-amodiaquine	(9) 6.8
	**Not informed (n = 7)**

Half of the health practitionners selected artemisinin derivatives because they believed these drugs were more effective than the currently used monotherapies, with limited adverse events (17.4%) compared to amodiaquine (Table 4). About 15% of the practitioners mentioned that observance of the treatment regimen is an important parameter. The choice of the prescription was also based on the following criteria: drug cost (10.6%), trade-named advertised drugs (4.5%) and patient's request (1.5%).

**Table 4:** Main selection criteria of artemisinin derivatives according to prescribers

***Selection criteria***	***Medical practitioners*** ***(n = 132), Percent***
Therapeutic efficacy	(67) 50.8
Few adverse events	(23) 17.4
Observance (few tablets)	(20) 15.2
Drug cost (lowest price)	(14) 10.6
Drug names	(6) 4.5
Patient's request	(2) 1.5

A total of 70.5% of the health professionals reported that they received the main source of information from medical visitors employed by pharmaceutical companies (Table 5).

**Table 5:** Main sources of information in urban health facilities

***Sources of information***	***Medical practitioners*** ***(n = 132) Percent***
Medical visitors	(93) 70.5
Health personnel	(17) 12.9
Medias (Radio, TV)	(9) 6.8
Ministry of public health	(9) 6.8
Medical reviews	(4) 3.0

## Discussion

The implementation of new treatment regimens faces several constraints, such as drug availability, drug cost and rational use. Moreover, in urban areas, the wild range of concurring anti-malarial drugs offered by the private sector represents the main limitations. Our investigation indicated that the first line combination therapy AS/AQ was not available at the national office of drug management at the time of the study. On the other hand, stocks of quinine tablets were available in both public and private drugstores and a large choice of anti-malarial drugs was offered by private drug stores, including artemisinin derivatives alone or proposed as co-blisters with amodiaquine or other anti-malarial drugs.

In addition, ACT is more expensive than chloroquine, SP or amodiaquine used in monotherapy, and improper and abusive use without proper diagnosis will have a direct clinical and economic impact [5-7]. Parasitological diagnosis of malaria is an important parameter leading to the appropriate use of anti-malarial drugs. The results of this study demonstrate that the diagnosis of malaria was mainly based on the presence of fever without further parasitological investigation. Such findings have been previously reported from many endemic countries and are no longer surprising [5]. The absence of parasitological diagnosis is based on (i) lack of laboratory equipment and quality control to ensure reliable results, on the sites of investigations, (ii) cost of diagnosis which increases consultation and drug expenses, and (iii) the assumption that fever is mainly related to malaria in most endemic countries. However, malaria control efficacy in low transmission settings, such as urban areas implies the use of malaria parasite detection [8].

The present study highlighted problems related to the implementation of ACT in Cameroon, including the lack of protocol guidelines and in-service training related to malaria treatment, as previously reported in Sudan [9] and Kenya [10]. The lack of awareness and information was an important criterion explaining the non-adherence. Most of health workers supported the use of AS/AQ if the drug is made available in proper formulations such as few tablets for adults and pregnant women, and oral (liquid) or rectal formulations for children. Because it is proposed as a syrup formulation, amodiaquine used in monotherapy was the first-line treatment in children. This behaviour is contradictory to the list of adverse events attributed to amodiaquine, and reported by adult patients and practitioners. Therefore, the adaptation of proper drug formulations appears to be a crucial treatment allocation factor.

The observations made during this survey have highlighted the lack of information regarding the transition from monotherapy to artemisinin-based combination therapies, as previously described in Zambia [11].

Furthermore, the availability of quinine tablets at low price, in routine prescription and the improper use of injectable forms could compromise the treatment of severe malaria. Quinine has already been identified as the most prescribed anti-malarial drug in Cameroon [12]. The present study revealed that the wide range of artemisinin derivatives used in monotherapy, especially paediatric forms, influenced the prescribing patterns of medical practitioners. Given the wide range of anti-malarial drugs offered in urban areas, and conflicting information given mainly by private medical visitors, a strong implementation of official sources of information is needed in order to promote suitable clinical practices and ensure proper and rational use of anti-malarial drugs.

The present study conducted in mid-2005 indicates that the use of AS/AQ was not implemented in urban areas of Yaoundé in Cameroon, and that the wide range of anti-malarials and trade-named artemisinin derivatives were not yet accepted by the practitioners. However, medical practitioners will support the use of the AS/AQ combination for the treatment of uncomplicated malaria, expecting that the drug will be properly formulated (paediatric forms, reduced number of pills to take) and offered at lower price. Training, information and participation of health professionals to decision making is a key to improve adherence to new protocol guidelines. Results from this study and other investigations conducted in other countries highlight the need to consider specific implementation guidelines, since most African countries have now adopted ACT. Beside drug availability, the acceptance of ACT by health professionals and the overall population should be considered early enough given previous and current practices and use of available anti-malarial drugs.

The situation had improved in 2007. Following WHO recommendations, a public release of the Cameroonian Ministry of Public Health announced that all anti-malarial drugs available in monotherapy should not be sold and used anymore in health facilities. Therefore, nationwide withdrawal of these drugs was performed in public and private pharmacies and drugstores, starting in January 2007. This measure might prove efficient if (1) the recommended AS/AQ formulation will be made available at the national level with adapted paediatric formulations, and (2) if the reasons why monotherapies are not recommended anymore are clearly explained to the overall population.

Furthermore, in February 2007, the cost of malaria treatment was officially reduced to 0.30 US$ and 1.3 US$ (1$ = 483 CFA, May 2008) in public health facilities, followed in April 2007 by private hospitals and pharmacies to reach a price between 0.48 US$ and 2.06 US$. The move by government to subsidize the cost of treating malaria should improve anti-malarial drug accessibility to the overall population.

## Conclusion

The use of AS/AQ was not implemented in 2005 in Yaoundé, despite the wide range of anti-malarials and trade-named artemisinin derivatives available. Nevertheless, medical practitioners are prepared to support the use of this combination, once it becomes available in a paediatric formulation, at an affordable price. Training, information and participation of health professionals in decision-making is one of the key elements to improve adherence to new protocol guidelines. Reporting data from the introduction of ACT could provide useful information to highlight the strength and weaknesses of ACT implementation programmes. Indicators from this study could be considered as the starting point for further evaluation of the availability and accessibility of ACT in Cameroon.

## Competing interests

There are no conflicts of interest in regard to this work. The views expressed in this paper are solely those of the authors. Trade names are used for identification only and do not represent endorsement by the National Malaria Control Programme of Cameroon.

## Authors' contributions

Study design: CS, MG, NVL, PM, DB, DM. Field work: CS, MG, PM. Analysis and interpretation of data: CS, MG, PM, NVL. Revision of the paper: CS, PM, DM. All authors read and approved the final manuscript.
